# Electronic Nose Testing Procedure for the Definition of Minimum Performance Requirements for Environmental Odor Monitoring

**DOI:** 10.3390/s16091548

**Published:** 2016-09-21

**Authors:** Lidia Eusebio, Laura Capelli, Selena Sironi

**Affiliations:** Department of Chemistry, Materials and Chemical Engineering “Giulio Natta”, Politecnico di Milano, Piazza Leonardo da Vinci 32, Milano 20133, Italy; lidia.eusebio@polimi.it (L.E.); selena.sironi@polimi.it (S.S.)

**Keywords:** odor detection, odor recognition, sensor array, accuracy, sensitivity, detection limit, odor concentration

## Abstract

Despite initial enthusiasm towards electronic noses and their possible application in different fields, and quite a lot of promising results, several criticalities emerge from most published research studies, and, as a matter of fact, the diffusion of electronic noses in real-life applications is still very limited. In general, a first step towards large-scale-diffusion of an analysis method, is standardization. The aim of this paper is describing the experimental procedure adopted in order to evaluate electronic nose performances, with the final purpose of establishing minimum performance requirements, which is considered to be a first crucial step towards standardization of the specific case of electronic nose application for environmental odor monitoring at receptors. Based on the experimental results of the performance testing of a commercialized electronic nose type with respect to three criteria (i.e., response invariability to variable atmospheric conditions, instrumental detection limit, and odor classification accuracy), it was possible to hypothesize a logic that could be adopted for the definition of minimum performance requirements, according to the idea that these are technologically achievable.

## 1. Introduction

Since Persaud and Dodd reported the first design of an electronic nose using chemical sensors and pattern recognition in 1982 [1], electronic noses have become more and more popular, thus leading several research groups over the world to study their development and application in several different sectors.

The most well-known, although often criticized, definition of electronic nose is the one by Gardner and Bartlett [2]: “An instrument, which comprises an array of electronic chemical sensors with partial specificity and an appropriate pattern-recognition system, capable of recognizing simple or complex odors”.

The most relevant developments and diffusion of electronic noses concern the food industry (e.g., [3,4]), involving different applications, such as process monitoring, shelf-life investigation, freshness evaluation, and authenticity assessment [5]. Other interesting and possible applications are proposed in fields such as cosmetics and pharmaceutical [6,7], medical diagnostics [8,9], and more recently, environmental [10].

However, despite initial enthusiasm and quite a lot of promising results, several criticalities emerge from many of these studies, regarding for instance sensor sensitivity/selectivity [11,12], interference with temperature and humidity [13], and time drift [14,15], which all negatively affect the quality of the signal produced by the sensor [16,17]. Other difficulties are highlighted concerning the suitability of data processing/classification algorithms [18,19], thereby ranging from the simplest to the most complex ones; validation is considered as a crucial aspect for effective pattern recognition, as well [20]. In a recent and extremely interesting paper by Boeker [21] such problems are all brought to light and discussed in depth. The main points of this realistic and critical analysis should be carefully considered by all those scientists willing to approach the growing world of “electronic noses”.

One of the most crucial points is that, terms such as “electronic nose”, “machine olfaction”, or “chemical sensing”, which are often used as synonyms to define this combination of chemical sensors and pattern recognition system [2], are misleading, as they intrinsically point to an analogy with the human sense of olfaction. It is important that scientists become aware of the fact that, no matter how sophisticated, an instrument comprising a limited number of sensors coupled with a signal-processing unit and statistical algorithms should not be interpreted as an electronic reproduction of the mammalian nose. In fact, the sense of olfaction is of such complexity that it was worth a Nobel Prize to Richard Axel and Linda Buck no longer ago than 2004 [21].

This observation should not even be interpreted as a negative attitude towards the development and use of the electronic nose: once its intrinsic limitations are well defined based on the awareness of the differences between this instrument and the human nose, then it is possible to effectively think of the design of specific instruments for different applications.

Based on these considerations, it appears clear that, for any application, it is of crucial importance to define specific objectives and limitations. Moreover, given the wide range of different electronic noses available on the market, often based on different sensor types, pattern recognition system, and/or functioning principles [4,22], it is also necessary to establish precise procedures aiming to verify the instrument suitability for the desired application and its utilization.

In general, a first step towards large-scale-diffusion of an analysis method is standardization. Given the intrinsic complexity of electronic nose features, standardization in this field should not concern the instrument hardware; instead, it should focus on the instrument performances, thus fixing for instance minimum performance requirements.

Of course, such minimum requirements have to be intended as application-specific; as an evident example, the classification uncertainty that can be considered tolerable in the case of analysis of fruit ripening should be considerably different from those associated with cancer diagnosis.

Among the possible uses of electronic noses, this paper deals with the specific example of environmental odor monitoring at receptors. In this field, standardizing electronic nose performances by defining a set of minimum requirements is of particular importance in order to guarantee that different instruments provide comparable results, thus making environmental odor impact assessment as objective as possible. As a matter of fact, electronic noses might represent an important tool for the direct determination of odor exposure at receptors, especially in those cases where odor dispersion modelling is hardly applicable [23]. As a support to these considerations, it is worth mentioning that the European Committee of Normalization (CEN) on Air Quality, i.e., the CEN TC/264, has recently constituted a specific working group for the writing of a European Standard on instrumental odor monitoring.

The aim of this paper is to describe the procedure adopted in order to evaluate electronic nose performances, for establishing minimum performance requirements. This procedure involved the testing of a commercialized electronic nose according to a set of criteria that were considered crucial for the instrument’s use as an environmental odor monitoring tool, as will be described more in detail in the next paragraph.

The novelty of this paper is not properly in the experimental methods adopted, nor in the specific results obtained, since this involved some conventional electronic nose testing procedure, but rather in the attempt to propose first scientifically acceptable criteria and, secondly, a rigorous testing procedure for the definition of minimum performance requirements. This procedure, although application-specific, might possibly be, with appropriate modifications, extended to other applications or to other e-nose-type instruments.

## 2. Definition of the Criteria for Electronic Nose Testing

As already mentioned, this paper limits its field of application to electronic nose testing for the performance evaluation of an electronic nose to be applied for environmental odor monitoring receptors [10].

As a matter of fact, odors are currently recognized as effective air pollutants and are therefore subject to regulation and control in many countries [24]. Nowadays several regulatory approaches all over the world are based on dispersion modelling [25]; however, there are several situations in which dispersion modelling is hardly applicable, for instance due to the difficulty of appropriately characterizing the odor emission rate from the source, because of its configuration (e.g., diffuse source) or the discontinuity of emissions [23]. Moreover, limitations of dispersion modelling should always be kept in mind, since a mathematical model—no matter how sophisticated—shall never be interpreted as the “truth” but at most as a good cause-effect representation of specific phenomena, entailing an intrinsic degree of uncertainty, and thus requiring validation [25,26].

For these reasons, the possibility to use electronic noses for the direct determination of odors at receptors, i.e., directly where their presence is lamented, is of growing interest (e.g., [27,28,29,30]).

Besides sensor stability, which is particularly relevant in case of prolonged use (i.e., continuous monitoring), the problems related to sensor sensitivity are crucial: On one hand, sensors have to be sensitive enough to detect the presence of odors at receptors, i.e., typically at very low concentrations, and on the other hand they must be robust towards the atmospheric variation of humidity and temperature connected to outdoor use [30].

Due to the intrinsic complexities related to this application, the idea of fixing (a set of) minimum requirements appears essential in order to provide a first step towards standardization, aiming to guarantee that the desired results are achieved, i.e., that odors are detected at a sufficiently low concentration, and that they are recognized with an acceptable degree of accuracy and repeatability.

Once the field of application is clearly identified, performance criteria should be defined on one hand in a sufficiently generic way as to be valid for the different sub-cases comprised in the desired application (e.g., in this case, for the monitoring of different emission types as for instance those related to wastewater treatment, oil & gas, foundries, livestock, etc.), and on the other hand also specifically enough to circumstantiate the procedure in a sufficiently precise way to make it repeatable and reproducible for different instruments and in different laboratories.

As already mentioned, this paper aims to propose an electronic nose testing procedure for performance evaluation aimed to the definition of minimum performance requirements in the specific field of environmental odor monitoring receptors.

The first step for the design of a suitable procedure is the identification of the performance aspects towards which minimum performances need to be guaranteed, which in turn will be the criteria towards which electronic nose will have to be tested.

In this study, the following aspects, which are quite common matters of sensor technology and analysis science, were deemed important for an effective applicability of an electronic nose to environmental odor monitoring:

- the capability of the instruments of giving “good”, i.e., repeatable, responses in conditions of variable atmospheric conditions (especially humidity and temperature), which are typical of the environmental outdoor use (“invariability of responses to variable atmospheric conditions”);
- the instrument sensitivity to odors, which should be high enough to detect the presence of odors at receptors, i.e., at a very diluted concentration (“detection limit”);
- the instrument capability of correctly recognizing the detected odors, by classifying them into a suitable olfactory class (“classification accuracy”).

Once the criteria for electronic nose testing have been established, the second step is the design of a suitable procedure to test the instruments towards those criteria.

The following part of this paper aims to give an example of the procedure adopted for electronic nose testing for the definition of minimum requirements relevant to the specific case of environmental odor monitoring at receptors. This involved an extensive experimental work carried out at the Olfactometric Laboratory of the Politecnico di Milano.

## 3. Materials and Methods

### 3.1. The Electronic Noses Used for the Tests

Two instruments were used for this work, both belonging to the EOS 507 model, produced by Sacmi s.c.a.r.l. and specifically developed for environmental applications (a detailed description of the instrument design and features is given in Dentoni et al., 2012 [30]).

**Figure 1:**
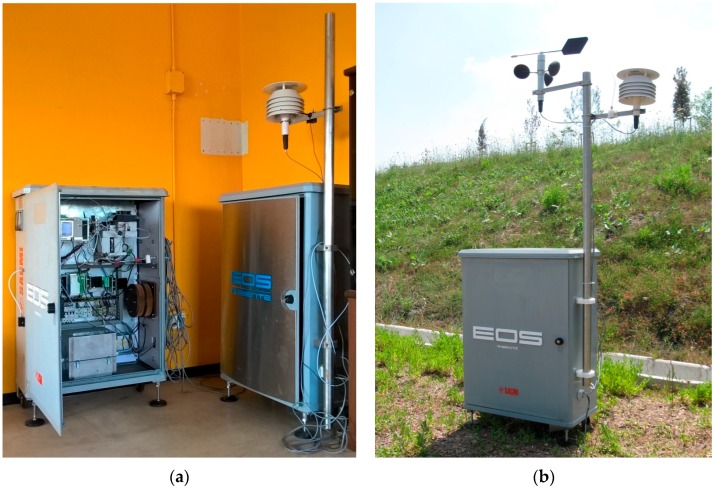
Electronic noses used for the tests in laboratory (**a**) and EOS 507 in the field (**b**).

Figure 1 shows the instrument interior (a) and its outer box with meteorological station as designed for outdoor use (b).

Each EOS507 electronic nose is equipped with six different MOS sensors, whose active layer typically consists of a layer of a single or a mixture of semi-conductive metal oxides (basically SnO_2_, but also WO_3_, In_2_O_3_, TiO_2_, NbO_5_), whose sensitivity can be modulated through the relative abundance of each metal oxide as well as by the addition of other components acting as catalysts, such as gold (Au), silver (Ag), or molibdenum (Mo) [27,30].

This instrument also has some innovative aspects with respect to other currently available electronic noses. First, the instrument is equipped with a system for the adjustment of the sample air humidity to a fixed value, calculated to optimize the instrument regulation capability based on the measured external ambient air humidity, through the parallel introduction of a neutral air stream, which is mixed with the sample stream before getting into the sensor camber [30]. This represents a very important improvement since, as already mentioned, varying atmospheric conditions—especially humidity—are one of the main critical aspects associated with the use of electronic noses for environmental odor monitoring, which typically involves their use outdoor.

Second, the reference substance constituting the instrument baseline is not neutral air, but a specific compound at known and constant concentration, named “standard”. This has the advantage of producing a more stable reference baseline, as little impurities in the “standard” flow do not produce considerable oscillations of the sensors’ conductivity as occurs with neutral air [30]. Moreover, the periodical analysis of the “standard”, i.e., of a reference substance at constant concentration, is a key feature to compensate sensor drift over time.

Finally, with respect to its predecessor EOS 835, in the EOS 507 a new option called “substance training” was introduced for the instrument training: when selected, the instruments dilutes the analyzed sample with different ratios of neutral air, as to analyze it automatically at different concentration levels, without requiring the manual preparation of samples at different concentrations as was done before [31]. In this case, the sample is typically analyzed at 10 different concentration levels starting from 10% of its concentration to 100%, with a step of 10%.

### 3.2. Identification of Target Compounds and Sample Preparation

In order to test electronic nose performances towards the criteria described in Section 2 it was first necessary to identify a set of pure compounds to be used as target compounds for the evaluations. The reason for the choice of using pure compounds is the will to guarantee repeatability and reproducibility of the procedure, in order to make it possible to compare the performances of different instruments tested in different labs or at different times and conditions. For this reason, it is useful to have the purity of the compounds used for the tests certified by the manufacturer.

The choice of the pure compounds is not trivial, since, according to the will of making the testing procedure as general as possible for different environmental applications, the target compounds to be tested should possibly be representative of a wide number of emission types. It was therefore decided to first identify the chemical compound families that are representative of environmental odor emissions. Based on an in-depth literature study, these turned out to be: alcohols, aldehydes/ketones, terpenes, sulphur compounds, and nitrogen compounds (e.g., [32,33,34,35,36,37]).

Five different compounds—one for each of the above mentioned families—were selected for electronic nose testing.

The selected compounds are reported in Table 1, together with their chemical family, the aggregation state, a description of the odor characteristics, and the typology of emissions in which these are typically present.

The methods employed for the preparation of the gas samples to be tested are related to the nature of the compound, i.e., if it is liquid or gaseous. The sample preparation methods are listed in the second column of Table 2.

When dealing with liquids, sample preparation was based on the headspace technique. After putting a known quantity of the liquid (30 mL) inside a bag for olfactometric sampling with a volume of 6 L, and then keeping its temperature controlled and constant (in this case 20 °C for 1 h) by means of a climatic chamber, liquid-vapor conditions are reached inside the sample at its storage temperature. By transferring the so obtained vapor phase inside another bag having the same volume, it is possible to create a gas sample with a constant and reproducible concentration “frozen” at the equilibrium concentration relevant to the storage temperature of the original sample. The evaluation of the equilibrium concentration of the vapor phase is easily determined by evaluating the vapor pressure of each compound at the storage temperature, which can be calculated bysemi-empirical equations with compound-specific coefficients such as those found in the Perry’s Chemical Engineers’ Handbook [46], which are reported in Table 2. This procedure was used for Ethanol, Acetone, and Limonene, for which the coefficients for the calculation of the vapor pressures are reported in Table 3.

$$C\left[\text{ppm}\right]=\frac{P_{ev}\left[Pa\right]}{P\left[Pa\right]}\cdot 10^{6}=exp\left[C1+\frac{C2}{T\left[K\right]}+C3\cdot ln\left(t\left[K\right]\right)+C4\cdot T{\left[K\right]}^{C5}\right]$$

For TMA, due to its different behavior with respect to the other liquid substances, a different procedure was adopted for the preparation of a gas sample at known concentration. As a matter of fact, pure TMA would be a gas at room temperature (its Boiling Point is 3.5 °C). It is commercially available as a liquid 4.2 M solution of TMA in Ethanol (33%). In this case, the evaluation of the equilibrium concentration is more problematic, because the activity of TMA in the liquid solution should be considered. For this reason, we decided to adopt a different procedure by inserting 1 μL of solution of TMA in a 6 L bag, which was then filled with neutral air. It can be calculated that this quantity of TMA is small enough with respect to the volume of 6 L that equilibrium conditions cannot be reached, therefore, by heating the sample in a climatic chamber, after 1 h, the whole liquid (1 μL of solution of TMA 33%) is vaporized inside the bag. This corresponds to 4.2 × 10^−6^ mol of TMA corresponding to a concentration of gaseous TMA inside the sample of ca. 17 ppm.

The H_2_S (gaseous) samples was obtained by dilution of a bag filled with H_2_S directly from bottle at known concentration. The bottle available in our laboratory had a concentration of 2020 ppm, by applying a dilution factor of 4096 by means of an Olfactometer Odornet TO8, a gas sample with an H_2_S concentration of ca. 0.5 ppm was obtained.

The concentration of the samples for electronic nose testing and the methods for their obtainment are resumed in Table 2. This sample preparation procedure exploiting the headspace of liquid compounds (except for H_2_S), despite being apparently intricate, guarantees a good repeatability and was chosen due to the availability, ease of supply and low cost of such liquid compounds. In the future, if a testing procedure based on the use of gas samples at fixed concentration should be standardized, then the sample preparation method simplicity and repeatability may be improved by the use of gas bottles at the desired concentration.

For the purpose of this study, which is testing the electronic nose towards odor, it is important to have the possibility to relate the analytical concentration of the test samples to their odor concentration. For this reason, it was necessary to evaluate the Odor Threshold (OT) concentrations of each of the target compounds, which is also reported in Table 2. Given the high variability of the OT that can be found in literature [47], this was done experimentally by calculating the ratio between the sample analytical concentration and its odor concentration measured by dynamic olfactometry [48]. Three to eight samples at different analytical concentrations were analyzed for each compound in order to make the OT determination more robust. The experimentally determined OT values were compared with those from Nagata [49], which are also reported as a reference in the only Italian Regional guideline on odor emissions, which is the guideline of the Region of Lombardy [50]. There is quite a good agreement between the experimental and the literature data, except for the OT of TMA, even though it should be highlighted that the OT reported by Nagata [49] is probably underestimated, since there are several other references assessing the OT of TMA in the order of magnitude of few ppb (e.g., [51,52]).

As will be discussed in the next sections, electronic nose testing towards the different target substances was performed using samples at known concentrations. The test samples were prepared at similar odor concentration ranges, and thus, due to the different OTs of the target compounds, not at similar analytical concentrations. This choice is justified by the fact that the aim of the electronic nose testing in this study is the evaluation of the instrument capability of measuring “odor”: therefore, it is important to express the sample concentration in terms of the effect that it provokes on humans in terms of olfactory sensation, i.e., its odor concentration, more than its analytical concentration. For this reason, in the following sections the test samples concentration is always expressed in terms of ou_E_/m^3^. As previously mentioned, once the odor concentration of a sample of one of the target compounds is fixed, its analytical concentration is univocally determinable by dividing the odor concentration by the OT concentration of the compound as reported in the second to last column of Table 2.

### 3.3. Experimental Methods

Specific tests were run in order to evaluate the electronic nose performances relevant to the criteria described in Section 2, i.e., invariability of responses to variable atmospheric conditions, detection limit, and classification accuracy.

As a general rule, electronic nose testing involves the following four phases:

- I: Creation of a “training” data set by means of the analysis of “training” samples of known quality and concentration;
- II: Creation of a “test” data set by means of the analysis of “test” samples of known quality and concentration;
- III: Execution of specific recognition tests for each aspect, whereby the electronic nose relates the test set to the training set and thus provides a classification of the test samples;
- IV: Evaluation of performance by evaluation by comparison of the classification values attributed by the electronic nose to the test set with respect to the true values.

In general, a recognition test involves to perform the classification of a match set based on a suitable training set, giving that one recognition value, i.e., in the case of qualitative recognition, an olfactory class, is attributed to every measurement of the match set. The output of the recognition test is therefore the attribution of an olfactory class to every measurement of the tested match set. The attributed olfactory class can then be compared with the effective (“true”) olfactory class relevant to each measure.

Performance evaluation can then be based on the determination of a so called “Accuracy Index” (AI), calculated as the ratio between the number measurements that were correctly classified and the number of total measurements [31,53]:

(1) $$\text{AI}\left(\%\right)=\frac{\sum R_{\text{corr}}}{\sum R_{\text{TOT}}}\times 100,$$

where R_corr_ are the correct recognitions and R_TOT_ the total recognitions.

As a further step, minimum performance requirements could therefore be established as minimum values of AI to be obtained in the recognition tests relevant to each of the considered aspects.

The next section will describe more in detail the experimental operations that were carried out in order to test the EOS 507 peformances in terms of invariability of responses to variable atmospheric conditions, detection limit, and classification accuracy according to this evaluation method.

## 4. Results

### 4.1. Invariability of Responses to Atmospheric Parameters

#### 4.1.1. Training Set (TS) Creation

The training set (TS) for the evaluation of the invariability of the EOS 507 responses to variations of the atmospheric conditions was created using the instrument “substance training” option, which allows the analysis of a sample at 10 different concentrations, from 10% to 100% of its concentration. The TS thus comprised 10 measurements for each compound in an odor concentration range between 80 and 1500 ou_E_/m^3^ (which means that if the “original” sample had a concentration of 1000 ou_E_/m^3^ then it was automatically diluted from 10%, i.e., 100 ou_E_/m^3^, to 100%, i.e., 1000 ou_E_/m^3^; the range 80–1500 ou_E_/m^3^ is given by the fact that the odor concentration of the “original” samples of the different compounds was comprised between 800 ou_E_/m^3^ and 1500 ou_E_/m^3^). As for variable atmospheric conditions, it was decided to consider temperature and humidity, which are the two meteorological parameters that most likely affect electronic nose sensor responses in outdoor environmental applications [10,13]. For this reason, the temperature (T) and the relative humidity (RH) had to be fixed. The T and RH values chosen for the training samples were 20 °C and 60%, respectively.

#### 4.1.2. Match Set (MS) Creation

The match set (MS) for the evaluation of the invariability of responses to temperature and humidity changes was created comprising 20 measurements for each target compound and for each different condition tested in an odor concentration range between 80 and 1500 ou_E_/m^3^.

The tests were executed by varying the T and the RH of the test samples, respectively. More in detail, eight different conditions were tested for each substance, considering two different test types: tests at fixed RH and variable T, and tests at fixed T and variable RH (i.e., four different conditions at fixed RH and variable T, and four different conditions at fixed T and variable RH were tested). The samples were analyzed considering one compound at a time, at increasing concentrations (from 10% to 100%).

Regarding the tests at fixed RH and variable T, the RH of the test samples was kept equal to the RH of the training samples (RH_MS_ = RH_TS_ = 60%), while the temperature was varied within a range of ±5 °C. Conversely, for the tests at fixed T and variable RH, the T of the test samples was kept equal to the T of the training samples (T_MS_ = T_TS_ = 20 °C), while the relative humidity was varied within a range of ±20%. The test ranges were chosen as to consider typical conditions that occur in ambient air.

#### 4.1.3. Recognition Tests

A scheme of the tests executed is reported in Table 4. Each of the 40 tests (eight conditions for each target compound) involved the recognition of a minimum number of 20 different measures, thus giving a total of over 800 recognitions.

The recognitions were based on the use of the whole TS, meaning that the electronic nose had to attribute each measure to one olfactory class among the five to which it was trained (i.e., the five target compounds). The principle of the test is to verify if the electronic nose is capable to recognize the target compounds correctly when the test sample has a different T or RH with respect to the training samples.

The results of the tests can be resumed in a table reporting the % of correct classifications (i.e., the “Accuracy Index” AI) for each of the target compounds and for each condition tested (Table 5). From the results reported in Table 5, it is possible to observe that Limonene, H_2_S, and TMA are recognized with satisfactory accuracy at all conditions tested. On the contrary, erroneous classifications occur mainly for Acetone and Ethanol, especially for the test samples at lower RH and T values than the training samples. By analyzing the single outputs of the recognition tests more in depth it is possible to observe that the main errors concern the “confusion” between Acetone and Ethanol, meaning that Acetone samples at low T and RH are erroneously recognized as Ethanol, and vice versa. This “confusion” between the two substances may be due to the fact that they present a higher chemical similarity to each other than the other compounds being testes, as they are both oxygenated organic compounds. This hypothesis was proven by subsequent tests conducted excluding Acetone (which means that Acetone samples were not analyzed neither for the TS nor for the MS creation), thus keeping only Ethanol as representative of the family of the oxygenated compounds. In this case, classification accuracy for the Ethanol test sample at 50% of RH of 92% (instead of 0%) was obtained.

In general, the different nature of the sensors that may be included in an electronic nose may result in a different sensitiveness towards different substances or families of substances. For this reason, we decided to evaluate the electronic nose capability to give correct responses when the T or the RH of the analyzed sample changes using an “overall” AI towards all considered target compounds, thus evaluating the AI calculated as the arithmetic mean of the AIs obtained for all target compounds in the tests with varying T and RH, respectively.

Based on the experimental results, the average AI obtained for the tests at constant T and variable RH turned out to be equal to 72%, while at constant RH and variable T this Index increases to 87%.

As a final consideration, it may be observed that the test ranges for temperature and humidity were chosen arbitrarily as to consider typical variations that may occur in ambient air. Future tests may consider wider ranges in order to increase results robustness.

### 4.2. Detection Limit and Classification Accuracy

#### 4.2.1. Training Set (TS) Creation

The training set (TS) for the evaluation of both the instrumental detection limit to the target odors and their classification accuracy was created comprising 20 measurements for each substance in an odor concentration range between 30 and 1000 ou_E_/m^3^. Since the instrument “substance training” option, which analyzes one sample at 10 different concentration levels by diluting it from 10% to 100% of its concentration, was used, this means that the TS creation involved the analysis of two samples for each target compound (10 measurements each), in the following concentration ranges: (i) 30–500 ou_E_/m^3^ (i.e., odor sample with concentration between 300 and 500 and ou_E_/m^3^, then diluted from 10% to 100%); and (ii) 70–1000 ou_E_/m^3^ (i.e., odor sample with concentration between 700 and 1000 ou_E_/m^3^, then diluted from 10% to 100%).

More in detail, this means that the samples of each substance were diluted comprising five odor concentration intervals, each of them comprising four measurements, according to the following distribution: four samples in the range 30–100 ou_E_/m^3^, four samples in the range 100–200 ou_E_/m^3^, four samples in the range 200–350 ou_E_/m^3^, four samples in the range 350–600 ou_E_/m^3^, and four samples in the range 600–1000 ou_E_/m^3^. The T and RH of the training samples were fixed at 20 °C and 60%, respectively, and regulated by means of a climatic chamber.

For this kind of evaluation, the TS shall include also a reference olfactory class “neutral air”, which corresponds to odorless air. In this case, the reference “neutral air” class was considered in the TS by adding 20 measurements of 20 different samples of ambient air collected outside the laboratory in different days.

#### 4.2.2. Match Set (MS) Creation

The match set (MS) for the evaluation of both the instrumental detection limit to the target odors and their classification accuracy was created comprising 10 measurements for each substance in an odor concentration range between 15 and 250 ou_E_/m^3^. Since the instrument “substance training” option was used also in this case, this means that the TS creation involved the analysis of one sample (i.e., 10 measurements) for each target compound having a concentration between 150 and 250 ou_E_/m^3^, which were then automatically diluted from 10% to 100%, given that the test samples were analyzed at increasing concentrations.

**Figure 2:**
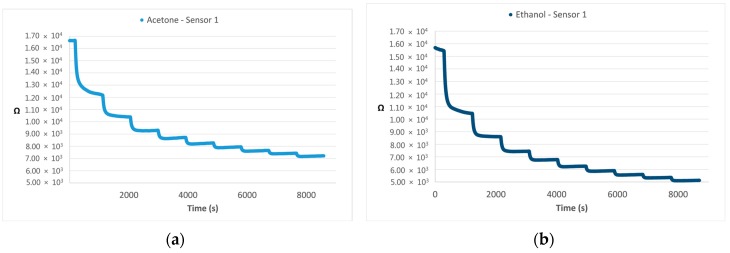
Examples of responses of sensor 1 in terms of resistance variation (Ω) to Acetone (**a**) and Ethanol (**b**) samples at 10 different dilution steps (from 10% to 100% of the original sample).

As an example, Figure 2 illustrates the responses of one sensor in terms of resistance variation (Ω)—which is the typical response of MOS sensors—to samples of Acetone (Figure 2a) and Ethanol (Figure 2b), both analyzed from 17 to 170 ou_E_/m^3^.

#### 4.2.3. Recognition Tests for the Evaluation of the Instrumental Detection Limit

Concerning the evaluation of the instrumental detection limit, for each test the electronic nose related the MS relevance to one substance to the TS relevance to the same substance plus the neutral air. As an example, if considering for instance Ethanol, then the TS for the recognition test comprised the 20 measurements of the Ethanol samples in the range 30–1000 ou_E_/m^3^ + the 20 measures corresponding to the neutral air samples, whereas the match set consisted of the 10 measurements of the Ethanol sample in the 15–250 ou_E_/m^3^ range.

This indicates that, for each of the five substances considered, the electronic nose had to classify the 10 “low concentration” (15–250 ou_E_/m^3^) measures based on the “high concentration” measures (30–1000 ou_E_/m^3^) of the same substance and the neutral air measures, thus giving a total of 50 recognitions (10 for each substance). The results of each test give that the 10 measures of the MS at increasing concentrations could be attributed either—incorrectly—to neutral air or—correctly—to the target compound. The correct classifications are expected to increase with the odor concentration of the test sample, giving that the odor concentration at which the sample classification switches from incorrect to correct can be identified as the detection limit (expressed in ou_E_/m^3^) for each of the considered substances. Of course, this detection limit may vary for each target compound, due to the different sensitiveness towards the different substances.

Table 6 reports a scheme of the tests executed for the evaluation of the instrumental detection limit towards the five target compounds as well as the results of the tests, which are expressed in terms of AI (i.e., % of correct classifications) obtained for each substance.

As can be seen, the instrument classified correctly all the measures in the tested odor concentration range, including the measures at the lowest odor concentration values (15–25 ou_E_/m^3^). This means that, according to the evaluation method proposed in this study, the instrumental detection limit of the EOS 507 towards the considered target compounds turned out to be <15–25 ou_E_/m^3^. Based on these satisfactory results, in the future the evaluation procedure could be further refined by testing the electronic nose behavior to samples having lower odor concentrations, e.g., 3–10 ou_E_/m^3^. When dealing with such low odor concentration values the significant uncertainty associated with the olfactometric measurements [54]—an error band between one-third and three-fold of an actual measurement value should be taken into consideration [55]—for instance by increasing the number of the test samples and/or by refining the OT concentration determination.

#### 4.2.4. Recognition Tests for the Evaluation of the Classification Accuracy

Finally, concerning the evaluation of the classification accuracy, the electronic nose related the MS relevant to one substance to a “complete” TS comprising the training measures relevant to all the compounds being considered (including the neutral air).

This means that the electronic nose had to classify the 10 “low concentration” (15–250 ou_E_/m^3^) measures of each substance based on the “high concentration” measures (30–1000 ou_E_/m^3^) of all substances, for a total of 50 recognitions. Therefore, since the TS comprised the measures relevant to all the target compounds and the neutral air, the electronic nose could attribute each test measure to neutral air or to any of the target compounds.

As an example, the 10 MS measures at increasing concentrations (15–250 ou_E_/m^3^) of Ethanol could be recognized either as Ethanol (correct classification) or misclassified, i.e., attributed to any other olfactory class (neutral air, Acetone, Limonene, H_2_S, or TMA).

Table 7 resumes the tests for the evaluation of the classification accuracy. The results are expressed in terms of AI (i.e., % of correct classifications) obtained for each substance. Also in this case, different AI values can be expected depending on the substance considered. In fact, very good results were obtained for all substances: 100% of correct recognitions for Ethanol, Limonene, and H_2_S; and 90% of correct recognitions (9 out of 10) for Acetone and TMA, which gives an “overall” AI of 96%.

## 5. Discussion and Conclusions

The aim of this paper was to describe the experimental procedure adopted in order to evaluate electronic nose performances, with the final purpose of establishing minimum performance requirements, which is a first crucial step towards standardization of the use of electronic noses for environmental odor monitoring.

Electronic nose performances were tested towards three criteria that were recognized as fundamental in the specific case of electronic nose application for odor impact assessment purposes, which involves the direct determination of odor exposure (i.e., both detection and recognition) at receptors [23,27]. The criteria considered therefore were basically the capability of the instruments of giving repeatable responses when atmospheric humidity and temperature are variable, their lower detection limit, and their capability of odor recognition. Of course, the rules for the definition of a minimum performance requirement should not be based on the performances of one specific electronic nose type, but on other, more “legislative” considerations instead, e.g., the error that may be considered as theoretically acceptable for an environmental monitoring instrument used for odor monitoring at receptors.

On the other hand, besides an ideal approach, for which it would be desirable to have instruments with 100% accuracy, precision, or repeatability, in a technical norm it is necessary to remain more practical and thus consider the state of the art of the proposed technology. This means that the limitations of the technology should be critically analyzed in order to expect performances that are effectively achievable by the best-performing instruments available on the market.

Based on the experimental results of the performance testing of two commercialized electronic noses produced by Sacmi [30] with respect to these three criteria, it is possible to hypothesize and propose a logic that could be adopted for the definition of minimum performance requirements, according to the idea that these are technologically achievable.

As a first example, according to the results presented in Section 4.1.3, a minimum performance requirement for the evaluation of the electronic nose response’s invariability to atmospheric conditions could possibly be fixed as two minimum AI values, one related to tests with variable RH and fixed T, and the other one to tests with variable T and fixed RH. In this case, AI values above 70% were obtained for both test types.

The evaluation of a minimum requirement regarding the instrumental detection limit is more difficult based on the results presented in Section 4.2.3, since the tested electronic nose proved its capability to recognize samples at odor concentrations as low as 15–25 ou_E_/m^3^ with 100% accuracy. It is possible that different results would have been obtained for lower odor concentrations, but these were not tested in this first test phase. For this reason, an improvement could be represented by lowering the odor concentration of the test samples as to get closer to the odor detection threshold concentration, which is by definition equal to 1 ou_E_/m^3^.

In this case, the minimum performance requirement could be defined as one (or more) minimum AI value that should be achieved above a given odor concentration threshold, as, just as an example, AI > 70% above 5 ou_E_/m^3^ and AI > 95% above 20 ou_E_/m^3^.

The classification accuracy achieved by the EOS 507 to the tested five compounds is very high, always being above 90%. Probably the definition of a minimum performance requirement could be more permissive, fixing minimum AI values around 70%–80%. As a further refinement of the minimum performance requirement relevant to the classification accuracy, it would be possible to imagine the definition of two different minimum requirements: one, more restrictive, for the discrimination of neutral air from odor, thus minimizing the possibility of false positive detections, and another one, less restrictive, for the discrimination of the different odors from each other. This proposal comes from the consideration that, in the case of environmental monitoring at receptors, as for example the case of the odor monitoring from a landfill at the house of a complaining citizen, it is generally more important to be accurate in the detection of the odor episodes, i.e., *when* the odor is present, than to be accurate in the classification of the detected odor into the olfactory classes that identify the landfill odor sources (e.g., landfill gas or leachate), i.e., *which* odor is present.

As a general consideration, it may be observed that in this study the electronic nose performance evaluation was always based on the determination of an overall AI, calculated as the arithmetic mean of the AI values obtained for each substance. This choice was based on the consideration that, as this paper focuses on the specific example of application of environmental odor monitoring receptors, environmental odors are typically complex mixtures of different substances. For this reason, it may be reasonable to consider the electronic nose capability to recognize odor as a whole, and not to focus on the sensitiveness towards single substances. However, rare cases may exist in which atmospheric emissions consist of one single compound (e.g., in the production of polymers the composition of the emissions into the atmosphere typically includes only the monomer), for which it could be more useful to evaluate the instrument performances in terms of a substance-specific AI. As a matter of fact, in such cases, the application of an electronic nose as monitoring tool may be unnecessary, and other analytical methods may be preferred.

Moreover, due to the complex nature of environmental odors, a further improvement of the proposed procedure could be to extend electronic nose testing not only to pure compounds, but also to standard synthetic mixtures of these compounds.

## Figures and Tables

**Table 1:** Selected target compounds for electronic nose testing.

Compound	Family	Aggregation State	Odour	Emission Typology
Ethanol	Alcohols	Liquid (≥99%, Sigma-Aldrich)	Alcohols	Composting, Waste treatment, Biogas [33,38,39]
Acetone	Ketones	Liquid (≥99.8%, absolute alcohol, without additive, Fluka)	Pungent	Wastewater treatment, Composting, Waste treatment, Biogas [38,39,40,41]
Limonene	Terpenes	Liquid ((R)-(+)-Limonene, ≈90%, sum of enantiomers, Sigma-Aldrich)	Citrusy	Composting, Waste treatment, Decomposition of plants, Water treatment [32,38,42]
Hydrogen sulfide (H_2_S)	Sulfur compounds	Gas (Bottle, certified concentration 2020 ppm, Sapio)	Rotten eggs, Persistent	Landfills, Sugar mills, Water treatment, Sludge treatment, Anaerobic decomposition, Anaerobic digestion [36,43]
Trimethylamine (TMA)	Nitrogen compounds	Liquid (Solution 31–35 wt. % in Ethanol, 4.2 M, contains toluene as stabilizer (Sigma-Aldrich)	Rotten fish, Persistent	Water treatment, Livestock, Rendering [44,45]

**Table 2:** Concentration of the target compounds in the test samples and odor detection thresholds.

Compound	Sample Preparation Method	Concentration Calculation	Concentration [ppm]	OT_exp_ [ppb]	OT_lit_ [ppb]
Ethanol	Headspace technique: 30 mL liquid in 6 L air, stored at 20 °C and 60% RH for 1 h, then separation of the headspace in another 6 L-bag	$C\left[\text{ppm}\right]=\frac{P_{ev}\left[Pa\right]}{P\left[Pa\right]}\cdot 10^{6}=exp\left[C1+\frac{C2}{T\left[K\right]}+C3\cdot ln\left(t\left[K\right]\right)+C4\cdot T{\left[K\right]}^{C5}\right]$	58000	2700 ± 630	520
Acetone			240000	29000 ± 4300	42000
Limonene		$C\left[\text{ppm}\right]=\frac{P_{ev}\left[mmHg\right]}{P\left[mmHg\right]}\cdot 10^{6}=exp\left[A-\frac{B}{C+T\left[{}^{\circ}C\right]}\right]$	2700	72 ± 16	38
Hydrogen sulfide (H_2_S)	Dilution 1:4096 from bottle at 2020 ppm	$C\left[\text{ppm}\right]=\frac{C_{bottle}}{dil.\;factor}$	0.5	0.25 ± 0.10	0.41
Trimethylamine (TMA)	Dilution 1:4096 from bottle at 2020 ppm	$C\left[\text{ppm}\right]=\frac{mol_{TMA}}{mol_{AIR}}\cdot 10^{6}$	17	1.0 ± 0.3	0.032

**Table 3:** Coefficients for the calculation of the vapor pressures of Ethanol, Acetone, and Limonene [46].

Compound	Coefficients				
	C1	C2	C3	C4	C5
Ethanol	74.475	−7164.3	−7.327	3.134 × 10^−6^	2
Acetone	69.006	−5599.6	−7.0985	6.224 × 10^−6^	2
	A	B	C		
Limonene	18.30108	5420.724	288.39		

**Table 4:** Tests executed for the evaluation of the electronic nose responses invariability to variable T and RH.

Tests at Variable RH				Tests at Variable T			
Test No.	Substance	RH (%)	T (°C)	Test No.	Substance	RH (%)	T (°C)
1	Acetone	40	20	21	Acetone	60	15
2	Acetone	50	20	22	Acetone	60	18
3	Acetone	70	20	23	Acetone	60	23
4	Acetone	80	20	24	Acetone	60	25
5	Ethanol	40	20	25	Ethanol	60	15
6	Ethanol	50	20	26	Ethanol	60	18
7	Ethanol	70	20	27	Ethanol	60	23
8	Ethanol	80	20	28	Ethanol	60	25
9	Limonene	40	20	29	Limonene	60	15
10	Limonene	50	20	30	Limonene	60	18
11	Limonene	70	20	31	Limonene	60	23
12	Limonene	80	20	32	Limonene	60	25
13	H_2_S	40	20	33	H_2_S	60	15
14	H_2_S	50	20	34	H_2_S	60	18
15	H_2_S	70	20	35	H_2_S	60	23
16	H_2_S	80	20	36	H_2_S	60	25
17	TMA	40	20	37	TMA	60	15
18	TMA	50	20	38	TMA	60	18
19	TMA	70	20	39	TMA	60	23
20	TMA	80	20	40	TMA	60	25

**Table 5:** Results of the tests for the evaluation of the electronic nose responses invariability to variable T and RH in terms of AI for each substance and for each condition tested.

	Tests at Variable RH				Tests at Variable T			
Substance	40%	50%	70%	80%	15 °C	18 °C	23 °C	25 °C
Acetone	0%	0%	77%	77%	94%	100%	100%	100%
Ethanol	7%	0%	100%	64%	0%	7%	100%	100%
Limonene	59%	95%	100%	92%	92%	83%	93%	100%
H_2_S	87%	91%	100%	100%	84%	100%	100%	86%
TMA	100%	100%	100%	100%	100%	100%	100%	100%

**Table 6:** Scheme and results of the tests for the evaluation of the instrumental detection limit towards the five target compounds.

Test No.	Substance	TS	MS	AI
1	Acetone	Acetone 30–1000 ou + Neutral Air	Acetone 15–250 ou	100%
2	Ethanol	Ethanol 30–1000 ou + Neutral Air	Ethanol 15–250 ou	100%
3	Limonene	Limonene 30–1000 ou + Neutral Air	Limonene 15–250 ou	100%
4	H_2_S	H_2_S 30–1000 ou + Neutral Air	H_2_S 15–250 ou	100%
5	TMA	TMA 30–1000 ou + Neutral Air	TMA 15–250 ou	100%

**Table 7:** Scheme and results of the tests for the evaluation of the classification accuracy.

Test No.	Substance	TS	MS	AI
1	Acetone	Complete TS: Acetone 30–1000 ou + Ethanol 30–1000 ou + Limonene 30–1000 ou + H_2_S 30–1000 ou + TMA 30–1000 ou + Neutral Air	Acetone 15–250 ou	90%
2	Ethanol		Ethanol 15–250 ou	100%
3	Limonene		Limonene 15–250 ou	100%
4	H_2_S		H_2_S 15–250 ou	100%
5	TMA		TMA 15–250 ou	90%
